# BCG Vaccine-Induced Neuroprotection in a Mouse Model of Parkinson's Disease

**DOI:** 10.1371/journal.pone.0016610

**Published:** 2011-01-31

**Authors:** Jing Yong, Goran Lacan, Hoa Dang, Terry Hsieh, Blake Middleton, Clive Wasserfall, Jide Tian, William P. Melega, Daniel L. Kaufman

**Affiliations:** 1 Department of Molecular and Medical Pharmacology, University of California Los Angeles, Los Angeles, California, United States of America; 2 Department of Pathology, University of Florida, Gainesville, Florida, United States of America; Centre de Recherche Public de la Santé (CRP-Santé), Luxembourg

## Abstract

There is a growing interest in using vaccination with CNS antigens to induce autoreactive T cell responses that home to damaged areas in the CNS and ameliorate neurodegenerative disease. Neuroprotective vaccine studies have focused on administering oligodendrocyte antigens or Copaxone® in complete Freund's adjuvant (CFA). Theoretical considerations, however, suggest that vaccination with a neuronal antigen may induce more robust neuroprotective immune responses. We assessed the neuroprotective potential of vaccines containing tyrosine hydroxylase (a neuronal protein involved in dopamine synthesis) or Copaxone® in CFA in the 1-methyl-4-phenyl-1,2,3,6-tetrahydropyridine (MPTP) mouse model of Parkinson's disease. Surprisingly, we observed that the main beneficial factor in these vaccines was the CFA. Since the major immunogenic component in CFA is *Mycobacterium tuberculosis*, which closely related to the bacille Calmette-Guérin (BCG) that is used in human vaccines, we tested BCG vaccination in the MPTP mouse model. We observed that BCG vaccination partially preserved markers of striatal dopamine system integrity and prevented an increase in activated microglia in the substantia nigra of MPTP-treated mice. These results support a new neuroprotective vaccine paradigm in which general (nonself-reactive) immune stimulation in the periphery can limit potentially deleterious microglial responses to a neuronal insult and exert a neurorestorative effect in the CNS. Accordingly, BCG vaccination may provide a new strategy to augment current treatments for a wide range of neuropathological conditions.

## Introduction

Vaccination with CNS antigens can induce autoreactive T cell responses that home to sites of injury in the CNS and can inhibit neuronal degeneration in different models of neurological disease and injury, including spinal cord and head injury, Parkinson's disease, Alzheimer's disease, amyotrophic lateral sclerosis, glutamate toxicity, and glaucoma (reviewed in [1], [2]). It is thought that immunization activates CNS-reactive T cells that enter the CNS, secrete neurosupportive factors (e.g., neurotrophins and brain-derived neurotrophic factor) and shift the phenotype of resident microglia to one that is more neurosupportive [3], [4]. The initial studies of this therapeutic strategy immunized animals with oligodendrocyte antigens such as myelin basic protein (MBP) (e.g., [5], [6]). Since autoimmunity to oligodendrocyte antigens can lead to a multiple sclerosis (MS)-like disease in experimental animals, subsequent studies of neuroprotective vaccines have focused on vaccinating with Copaxone® (e.g., [7], [8], [9], [10]). Copaxone® is a mixture of synthetic polypeptides composed of four amino acids in a random sequence dissolved in an aqueous solution. Frequent Copaxone® injection induces regulatory T cell responses that have partial cross-reactivity with myelin antigens [11] and this treatment has been approved as a therapy for relapsing-type MS. The vast majority of neuroprotective vaccine studies in animal models of neuropathological disorders have, however, administered myelin antigens or Copaxone® in complete Freund's adjuvant (CFA), an adjuvant that is unsuited for human use. There have been few reports of adjuvant-free Copaxone® having beneficial effects in animal models of neuropathological disorders other than MS [12], [13].

Our previous studies of antigen-based vaccine therapies for inhibiting autoimmune disease have shown that the ability of a vaccine to induce protective T cell responses depends critically on which self-antigen is administered [14], [15], [16]. This is because each self-antigen has a unique expression pattern and impact on T cell self-tolerance induction. Accordingly, self-antigens have different immunogenicities and should vary in their ability to induce neuroprotective T cell responses. Random copolymers as Copaxone® may not be optimal immunogens for inducing neuroprotective T cell responses since only a small portion of the induced T cell response may be capable of cross-reacting with CNS antigens. Hence, further studies are needed to examine how the nature of the antigen used in neuroprotective vaccines affects the efficacy of the treatment.

Current treatments for Parkinson's disease (PD) temporarily ameliorate its symptoms but do not slow progressive loss of dopaminergic neurons. Accordingly, new approaches to slow the degeneration of the nigrostriatal dopaminergic system are urgently needed. It is thought that oxidative stress, protein nitration and activated microglia contribute to the loss of dopaminergic function in human PD [17], [18], [19], [20]. Additionally, there is a growing appreciation that CD8^+^ and CD4^+^ T cells significantly infiltrate the SN of patients with PD [21], [22]. All of these potentially pathogenic factors are elicited by treatment with the neurotoxin MPTP [23], [24], [25]. The MPTP mouse model of PD has therefore been extensively used to assess neuroprotective strategies. Several studies have shown that vaccination with oligodendrocyte antigens or Copaxone® in CFA preserves dopaminergic neurons in MPTP-treated mice [10], [26], [27], [28]. These studies, however, did not determine whether the vaccine-induced immune responses limited the initial nigrostriatal dopamine system damage and/or promoted long-term neurorestoration.

We began our studies asking whether vaccination with tyrosine hydroxylase (TH), a neuronal protein involved in dopamine synthesis, could protect striatal dopaminergic neurons to a greater extent than Copaxone® in the MPTP model of PD in mice. Contrary to our expectations, we observed that immune stimulation by the CFA adjuvant, regardless of the emulsified antigen, appeared to be the major neuroprotective factor. CFA contains inactivated *Mycobacterium tuberculosis* in mineral oil and is unsuitable for human use. The BCG vaccine developed against childhood tuberculosis contains live attenuated *Mycobacterium bovis* that is closely related to *Mycobacterium tuberculosis*, and has been administered safely to billions of individuals since the 1920s [29], [30]. We describe the neuroprotective effects of BCG vaccination in the MPTP mouse model and discuss possible underlying mechanisms. Our results suggest that general (nonself-reactive) immune stimulation in the periphery may provide a new strategy to help slow disease progression in some neurodegenerative diseases.

## Materials and Methods

### Animals

All studies were approved by the UCLA Chancellor's Animal Research Committee (approval #2000-023-33). Male C57BL/6J mice (Jackson Laboratory), 8–10 weeks in age were used in all studies.

### Antigens for vaccination

We subcloned a mouse TH cDNA into a bacterial expression vector (pET-15b, Novagen) which directed the expression of TH with a short polyhistidine tag on it's carboxy terminus, and purified the TH from IPTG-induced recombinant *E.coli* inclusion bodies using nickel affinity chromatography. The eluted proteins were further separated using preparative SDS-PAGE and the band corresponding to TH was excised, electroeluted and the protein dialyzed into saline and frozen at −80° C until use. Copaxone® was purchased from TEVA Pharmaceutical Industries. BCG (TheraCys®) was purchased from Sanofi Pasteur.

### Vaccinations

Mice were vaccinated with the indicated antigen (100 µg) in CFA (1 mg/ml mycobacteria) in 0.10 ml subcutaneously at base of tail. Other mice were vaccinated with live BCG (2×10^7^ cfu) intraperitoneally (i.p.).

### MPTP treatment

Ten days after vaccination, mice were injected with 20 mg/kg MPTP i.p. for 5 consecutive days as previously described [31], [32].

### WIN binding assays

[^3^H]WIN 35,428 (WIN, 87.0 Ci/mmol) was obtained from Perkin Elmer Life Sciences (Boston, MA). All other chemicals were obtained from Fisher Scientific (Pittsburgh, PA). Mice were killed by decapitation; the brains were removed and sectioned into 1-mm coronal slices with a mouse brain mold on ice. Left and right striatal tissues were dissected out on an ice-cold stainless steel plate to constitute 2 samples which were frozen in dry ice and stored at −80°C until analysis. One striatal sample was used to measure [^3^H]WIN 35,428 binding to the dopamine transporter (DAT) in a homogenate preparation, according to our previously published methods [33].

### HPLC-EC analysis of dopamine content

The remaining striatal sample from individual mice was homogenized by ultrasonication in 0.5 ml ice-cold 0.2M HClO_4_ containing 0.15% (w/v) Na_2_S_2_O_5_ and 0.05% (w/v) Na_2_EDTA and centrifuged at 14,000 rpm for 15 min at 4°C. The supernatants were filtered through a 0.2 µm PTFE filter; the pellet was analyzed for protein content. An aliquot of 20 µl filtrate was used for HPLC-EC analysis of dopamine (DA) as previously described [34].

### Stereological analysis of Iba1+ microglia cell and TH+ cell numbers in the substantia nigra

Serial transverse sections of mouse midbrain were cryosectioned (30 µm using a Leica CM1850) and every fourth serial section was immunostained with anti-Iba1 (1∶500, WAKO Chemicals) and visualized by using the chromogen diaminobenzidine. The number of Iba1+ microglia in the substantia nigra pars compacta (SNc) were counted using an Olympus BX40 microscope and StereoInvestigator software (MicroBrightField). In brief, the two parts of the SNc were delineated on each section with 4× objective lens by an investigator who was blinded to the treatment. The counting was performed using optical fractionator formula [35] and a 100× oil lens. Representative images of Iba1+ cells were taken using a 100× oil lens on an Olympus BX50 microscope and an Insight Firewire 14.2 Color Mosaic digital camera (Diagnostic Instrument Inc.) using Metamorph 8.0 software.

Stereological analysis of TH+ cells in the SNc was conducted in a similar manner, with every fourth serial section immunostained with anti-TH antibody (1∶1000, Calbiochem-Novabiochem Corp., San Diego) and visualized by using the chromogen diaminobenzidine.

### Statistical analysis

All values are expressed as group mean ± SEM. Differences among groups were analyzed by one-way ANOVA, followed by post hoc Student's t-test for pair-wise comparison. The null hypothesis was rejected at p≤0.05.

## Results

### CFA is the major beneficial component of neuroprotective vaccines in the MPTP mouse model

Studies of neuroprotective vaccines have focused on using Copaxone® since it induces protective immune responses that cross-react with myelin antigens and because it is in clinical use for treating MS. We wanted to test whether immunization with a dopaminergic neuron antigen might have a more beneficial effect in the MPTP mouse model of PD, since this should direct vaccine-induced T cells to the brain areas that were damaged by MPTP treatment and that slowly degenerate in human PD. We chose tyrosine hydroxylase as a test antigen because it is involved in dopamine synthesis and is predominantly expressed in striatal dopaminergic neurons. We isolated TH from recombinant E. coli inclusion bodies, and purified it using affinity chromatography and preparative SDS-PAGE as described in Materials and Methods. Gel analysis of the purified TH is shown in Supplement Figure S1.

Since it takes 10–14 days for vaccine-induced immune responses to peak, and MPTP has a very immediate toxic effect, we immunized mice with TH or Copaxone® in CFA (TH/CFA and Copaxone®/CFA, respectively) 10 days before MPTP treatment. A group of control mice received only saline. The animals were sacrificed 21 days after the last MPTP treatment, which is a relatively long time point for such studies, because we wanted to test for potential neurorestorative effects of vaccination. As an initial read-out of the vaccine's ability to preserve dopaminergic system integrity, we measured [^3^H]WIN 35,428 (WIN) binding to DAT in mouse striatal homogenates.

**Figure 1:**
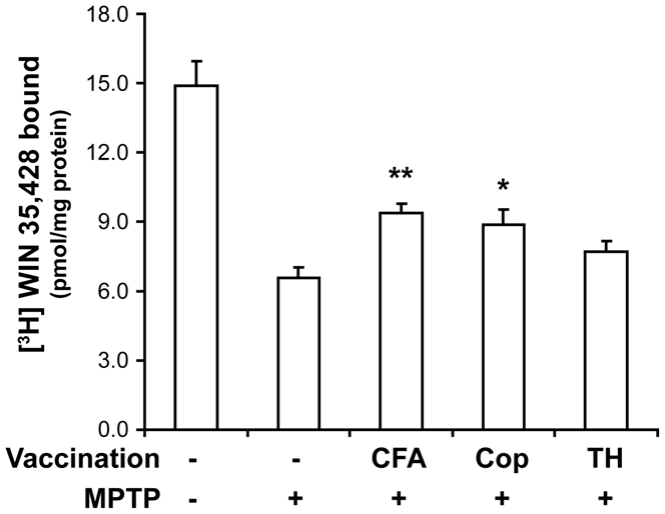
Vaccination with CFA preserves striatal DAT binding in MPTP-treated mice. Groups of mice were vaccinated with CFA (alone), Copaxone®/CFA or TH/CFA prior to MPTP treatment. Twenty one days after the last MPTP treatment, striatal DAT levels were assessed using a [^3^H]WIN-35,428 binding assay. Data shown are mean [^3^H]WIN-35,428 binding±SEM to striatal homogenate. N = 6–8 mice/group. *p<0.05, **p<0.01 vs. MPTP (alone) by Student's t-test.

We found that the mean DAT WIN binding levels were higher in striata from MPTP-treated mice that received CFA, regardless of whether they received CFA alone, TH/CFA, or Copaxone®/CFA, compared to that in unvaccinated MPTP-treated mice (Fig. 1). Specifically, compared to unvaccinated MPTP-treated mice, the levels of striatal WIN binding were 43% higher in MPTP-treated mice that received CFA alone (p<0.01) and 34% higher in mice that received Copaxone®/CFA (p<0.05). The level of striatal WIN binding was 17% higher in MPTP-treated mice that received TH/CFA, but this was not statistically significant. These results argue against our initial hypothesis that a neuronal self-antigen may provide a more efficacious neuroprotective vaccine. Rather, the results suggest that peripheral immunostimulation by CFA was the major beneficial factor.

### CFA vaccination promotes neurorestoration

It is possible that immune responses elicited by CFA vaccination limited MPTP's direct effects or promoted the subsequent restoration of dopaminergic neuron integrity. We therefore performed a more detailed study of the effects of CFA immunization on DAT levels 4 and 21 days after the last MPTP treatment.

**Figure 2:**
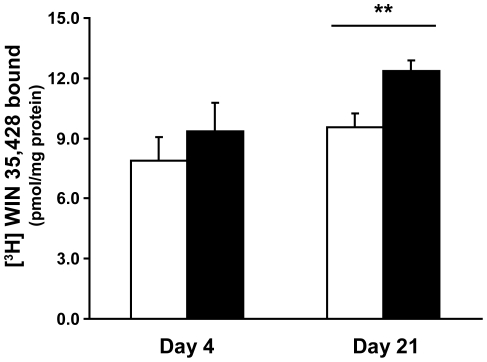
Higher striatal DAT binding in CFA-vaccinated mice 21 days post-MPTP-treatment. A control group of non-vaccinated mice received MPTP only (open bars) and another group of mice received BCG prior to MPTP treatment (black bars). Mice were sacrificed 4 or 21 days after the last MPTP treatment and their striatal DAT binding capacity was determined. Data shown are [^3^H]WIN-35,428 (mean±SEM) binding to striatal tissue homogenate (n = 7–8 mice/group) in two independent experiments. *p = 0.05, **p<0.01 by two-way ANOVA.

Groups of mice were vaccinated with CFA (alone) or saline, and 10 days later were given MPTP for 5 consecutive days. Four days after the last MPTP treatment, the mean levels of striatal WIN binding were 18% higher in the CFA treated group than in unvaccinated MPTP-treated mice, but this increase was not statistically significant (Fig. 2.). This suggests that CFA vaccination did not differentially affect the uptake, distribution or metabolism of MPTP and that CFA-induced immune responses have little or no ability to limit the acute toxicity of MPTP.

We also examined similarly treated mice 21 days after the last MPTP treatment. We found that the mean levels of striatal DAT WIN binding was 29% higher in CFA vaccinated mice that received MPTP, compared to unvaccinated MPTP-treated mice (Fig. 2, p<0.01). These data again demonstrate the beneficial effects of CFA (alone) treatment in our model. Additionally, the increase in striatal WIN binding observed in vaccinated vs. unvaccinated MPTP-treated mice from 4 to 21 days post-treatment (18% and 29%, respectively), suggests that CFA treatment promoted a greater rate of neurorestoration. Indeed, 21 days after MPTP treatment, only CFA-treated mice displayed a significant increase in striatal DAT WIN binding compared to levels 4 days post-treatment, suggestive of a neurorestorative effect.

### BCG vaccination partially preserves striatal DA and DAT in MPTP-treated mice

CFA is unsuitable for human use, but its main immunogenic component, inactivated *Mycobacterium tuberculosis*, is closely related to the live attenuated *Mycobacterium bovis* used in the BCG vaccine against childhood tuberculosis. We hypothesized that the peripheral immune responses induced by BCG immunization may also be neuroprotective.

**Figure 3:**
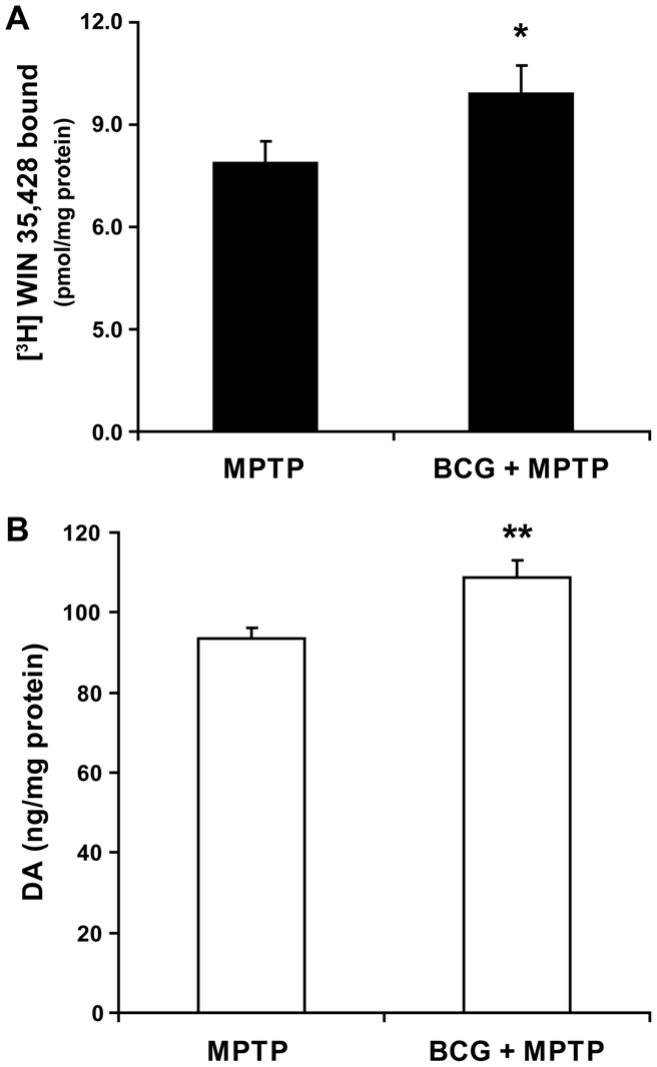
BCG vaccination preserves DAT-ligand binding and DA levels in MPTP-treated mice. Mice were vaccinated with BCG, or administered saline, prior to MPTP treatment. Twenty one days after the last dose of MPTP we analyzed striatal [^3^H] WIN-35,428 binding (A) and DA levels (B). Results from two independent experiments were pooled (total 16–18 mice in each group). Data shown are mean±SEM. *p<0.05, **p<0.01 by two-tailed t-test.

C57Bl/6 mice were immunized with BCG and 10 days later they, and a control group of unvaccinated mice, received MPTP. Twenty one days later, their striatal WIN binding levels were measured. Mice vaccinated with BCG had significantly higher levels of WIN binding than MPTP controls (26% higher, P = 0.05, Fig. 3A). In addition, striatum from mice vaccinated with BCG also had significantly higher DA content (16% higher than MPTP controls, p<0.01, Fig. 3B). Thus, BCG vaccination had a significant beneficial effect on both striatal DA content and DAT ligand binding levels.

### BCG vaccination prevents an increase in the number of activated microglia in the substantia nigra following MPTP treatment

Previous studies have shown that the number of microglia increases rapidly in the striatum after MPTP treatment and play an active role in MPTP-induced nigro-striatal system damage [24], [36], [37], [38], [39], [40], [41],[42]. Inflammatory type microglia are considered detrimental to neuron survival after a neuro-toxin insult [36] and blockade of microglia activation was neuroprotective in the MPTP mouse model of PD [24], [37], [38], [39], [40], [41], [42].

**Figure 4:**
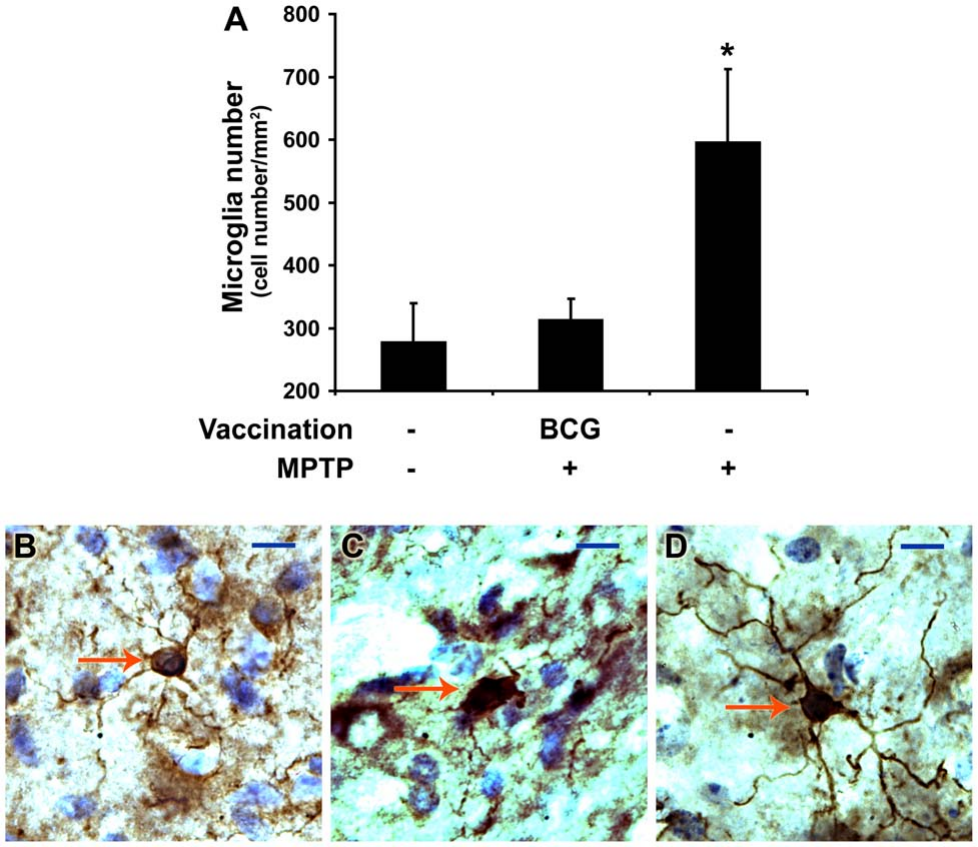
BCG vaccination prevents the MPTP-induced increase of microglia in the substantia nigra. Serial midbrain sections from mice that were, or were not, treated with BCG or MPTP were stained with anti-Iba1 antibody. A). Quantitative analysis of Iba1+ microglia. Data are represented as mean±SEM. (*p<0.05 by two-tailed t-test) N = 4–6 mice/group. B). Representative micropraghs of Iba1+ cells in the substantia nigra of mice treated only with saline (B), MPTP only (C), and BCG+MPTP (D). Note small cell body and long ramified processes of the Iba1+ cells in the saline (B) and BCG-vaccinated MPTP-treated mice (D), in contrast to the larger cell body with few processes in MPTP treated mice (C). Scale bar = 10 µm.

To examine whether BCG vaccination also affected the microglial reaction to MPTP toxicity, we treated other groups of mice with BCG or saline prior to MPTP treatment and counted the number of microglia in their midbrains three days post-MPTP treatment. We found that the number of Iba1+ microglia cells was significantly greater in animals that received MPTP compared to that in mice that received only saline (Fig. 4A), as also reported by others (e.g., [10], [24], [37]). In contrast, the Iba1+ cell number in SNc of BCG-vaccinated mice that received MPTP was similar to that in mice that only received saline (Fig. 4A). We also observed that the Iba1+ cells in the unvaccinated MPTP-treated mice had large cell bodies with only a few short thick processes, a morphology associated with microglia activation. In contrast, the Iba1+ cells in mice that received BCG before MPTP treatment had small cell bodies with long-fine processes similar to those in saline-treated control mice, suggesting a resting state (representative images in Figs 4B–D) [10], [13]. Thus, BCG vaccination prevented the MPTP-induced increase in the number of activated microglia in the SNc, suggesting that general immune stimulation in the periphery can limit CNS microglia responses to a neuronal insult.

At 21 days post-MPTP, stereological analysis revealed that the number of TH+ cells in the SNc of animals that received BCG was on average 6% greater than that in mice that received only MPTP (6400±424 vs. 6034±321, respectively), although this was not statistically significant (n = 6 and 7 mice/group, respectively).

## Discussion

Vaccination with CNS antigens has beneficial effects in a number of different animal models of neurological disease and injury. This strategy is based on inducing CNS-reactive T cells which home to areas of damage and exert beneficial effects locally in a process termed “protective autoimmunity” [1]. Early studies of neuroprotective vaccines administered myelin antigens, which raised safety concerns because of their potential for inducing a MS-like disease. Subsequent studies used Copaxone® which has some resemblance with MBP, and in its aqueous form is approved for MS treatment. Almost all these studies, however, used CFA as an adjuvant and often did not report on the effects of CFA alone. The studies that did examine CFA (alone) often reported that these treatments had some beneficial effect, although of lower magnitude than the myelin antigen or Copaxone® in CFA [10], [26], [43], [44].

Contrary to our initial expectations, we found that immunization with a dopaminergic neuron antigen did not provide a greater beneficial effect. Rather, CFA itself appeared to be main factor associated with higher levels of striatal WIN binding in vaccinated MPTP-treated mice. CFA treatment did not significantly alter the level of striatal DAT WIN binding 4 days after MPTP treatment, suggesting that CFA-induced immune responses cannot limit the acute toxicity of MPTP. Twenty one days post-MPTP treatment, however, the average levels of striatal DAT WIN binding in CFA treated MPTP-treated mice was significantly greater than that in unvaccinated MPTP-treated mice. The ratio of striatal WIN binding in vaccinated mice versus unvaccinated MPTP-treated mice increased from 4 to 21 days, suggesting that the CFA-induced responses promoted a greater rate of neurorestoration. CFA-treated mice, but not unvaccinated mice, had significantly higher striatal WIN binding 21 days vs. 4 days after MPTP treatment, indicating a neurorestorative effect.

Based on the neuroprotective effects of CFA, we turned to testing BCG vaccination. Potential advantages of BCG vaccination include not only its established safety record over many decades of worldwide use in humans, but also that the attenuated BCG slowly replicates in the vaccinated individual, inducing immune responses over many months. Accordingly, BCG vaccination could provide a long-term source of neurosupportive immune responses.

We observed that BCG vaccination significantly preserved striatal DAT WIN binding and DA content compared to that in unvaccinated MPTP-treated mice. Usually, MPTP treatment causes an increase in the number of nigral microglia, which may be due to resident microglia replication, or the influx of bone-marrow-derived cells (BMDCs, a macrophages or microglia precursors) from the periphery [45], . The increased number of activated microglia in the SNc is thought to contribute to MPTP-induced nigrostriatal system damage (e.g., [24], [37], [38], [39], [40], [41], [42], [46], [47].). Consistent with those reports, we observed that MPTP-treated mice displayed a greater than 2-fold increase in the number of Iba1+ cells in their SNc. In contrast, mice that were treated with BCG prior to MPTP treatment had a similar number of SNC Iba1+ cells to that in saline-treated control mice. In addition, in BCG treated mice, the nigra microglia had small cell bodies and long ramified processes, indicating a resting state. Such microglia are thought to exert neurosupportive functions by their abilities to produce neurotrophins and eliminate excitotoxins [13], [48]. These observations parallel previous assessments of microglia in MPTP-treated mice that received an adoptive transfer of spleen cells from Copaxone®/CFA vaccinated mice [10]. However, our results show that peripheral BCG-induced immune responses are sufficient to almost completely inhibit the MPTP-induced increase in activated microglia number in the SNc. Conceivably, by circumventing the MPTP-induced increase in activated microglia and the accompanying proinflammatory milieu, the surviving dopaminergic neurons were better able to recover function in BCG-treated mice. Further studies will be necessary to establish how the marked alterations in microglia morphology and activation affect long-term nigrostriatal dopamine system integrity.

Proposed mechanisms for neuroprotective vaccines have been contradictory in regard to whether Th1, Th2, Th3 and/or Treg cells play beneficial or pathogenic roles. Some of these differences may be due to the different disease models studied. Focusing on studies of immune-mediated protection in the MPTP mouse PD model, recent studies have pointed to CD4^+^ T cells as playing a key role in neurodegeneration [22]. Th17 cells recognizing nitrated α-synuclein can exacerbate MPTP-induced neuronal cell loss, but can be held in check by Tregs [41], [49]. The immune responses elicited by CFA and BCG have been extensively studied and they are both potent inducers of IFNγ-secreting Th1-type CD4^+^ T cells and activators of antigen presenting cells (APCs) [50], [51], [52]. IFNγ is known to antagonize the development of Th17 cells [53], [54] and can induce apoptosis of self-reactive T cells [55]. Additionally, BCG or *Mycobacterium tuberculosis* infection induces Tregs that proliferate and accumulate at sites of infection, which contribute to limiting inflammatory responses and tissue damage during infection [56], [57], [58], [59]. Accordingly, the Th1 and Treg responses may have suppressed the priming and expansion of Teffector cells following MPTP treatment. It is possible that the robust T cell responses to BCG also created greater T cell competition for APC that reduced the priming of Teffector cells in the periphery.

Another possible protective mechanism is that the active BCG infection in the periphery diverted Teffectors, macrophages and BMDC microglia precursors from entering the CNS after MPTP treatment. Previous studies in the experimental autoimmune encephalomyelitis (EAE) model have shown that infection with BCG 6 weeks before the induction of EAE diverts activated myelin-reactive CD4+ T cells from the CNS to granulomas in the spleen and liver [60]. This diversion was not due to cross-reactivity between BCG antigens and encephalitogenic proteins [61]. Evidently, the peripheral inflammatory lesions non-specifically attracted Teffectors that blunted the development of EAE. Interestingly, in clinical trials, MS patients immunized with BCG had a 57% reduction of lesions as measured by MRI [62], [63]. Thus, there is some clinical evidence that BCG treatment can suppress a neurodegenerative autoimmune response.

Based on our observations that MPTP did not increase microglia number and that microglia were in a resting state in the nigra of BCG-vaccinated mice, it is possible that the BCG treatment circumvented the activation and replication of resident microglia, diverted macrophage or BMDC microglia precursors from entering the CNS, and/or induced some efflux of macrophage-type cells to the periphery. Another possible protective mechanism is that the long period during which the attenuated BCG slowly replicates in the host causes a long-term increase in the levels of circulating immune factors (e.g., cytokines, chemokines or other factors), many of which can enter the CNS [64]. These immune factors may have limited microglia activation and proliferation, the influx of peripheral macrophages or microglia precursor cells, or had a supportive effect on neurons in the area of injury. Further testing is required to distinguish among these possibilities.

There are additional lines of evidence that peripheral immune responses can modulate the CNS milieu. Many studies have shown that treatment of pregnant rodents with immuno-stimulants such as lipopolysaccharide, polycytidylic acid, turpentine or viral infection, cause the offspring to have behavioral abnormalities (reviewed in [65], [66]). It is thought that the maternal immune responses to these treatments can alter neurodevelopment in the fetus. These studies provide further evidence that peripheral immune responses can modulate the CNS milieu independently of CNS-reactive T cells.

While the exact mechanisms of BCG neuroprotection in the MPTP mouse model remain to be elucidated, our results suggest that peripheral BCG-induced immune responses can exert neuroprotective effects independent of CNS antigen-specify. This represents a paradigm shift from the current notion that neuroprotective vaccines work by inducing protective T cell autoimmunity that acts locally in damaged areas in the CNS. It will be of interest to transfuse GFP-marked BMDC and T cells into mice prior to BCG and MPTP treatments in order to further study BCG's protective mechanisms.

Is there epidemiological evidence that BCG vaccination could be neuroprotective? BCG vaccinations were discontinued in the USA in the 1950s largely because of the low incidence of TB and the vaccine's incomplete protection. However, BCG vaccination is still given to infants and children in many countries. Adults who were BCG vaccinated as children have little/no protection from TB. Because BCG vaccine effects have greatly diminished by middle age, we would not expect to find a relationship between childhood BCG vaccination and PD incidence. Moreover, the BCG vaccine-mediated protection from TB relies on a small population of memory T cells that is quiescent and that only expands after re-exposure to TB. Since PD patients are not normally exposed to active TB, their few BCG-reactive memory T cells should be quiescent and would not be a source of neuroprotective factors.

While neuroprotective vaccines cannot correct basic intrinsic neuronal deficits, they may alter the CNS environment to be more neurosupportive so that neurodegeneration and secondary damage to neurons progresses at a slower rate. Conceivably, BCG-induced neuroprotective immune responses will be more beneficial in a slowly progressing disease, as in human PD, than in the acutely neurotoxic MPTP model we have studied.

In summary, our data show that BCG vaccination, which is safe for human use, can preserve striatal dopaminergic markers. This strongly supports the notion that peripheral immune responses can be benifical in neuropathological conditions. Second generation recombinant BCG vaccines, which have greater immunogenicity and are expected to elicit enhanced immunity against TB, are now being tested in clinical trials. Some new recombinant BCG strains express a human cytokine to boost desired immune responses. It will be of interest to test whether different recombinant BCG strains can enhance the vaccination's neuroprotective effects. Further studies of how peripheral immune system responses can modulate neurons and glia in the CNS may provide new therapeutic strategies to safely slow neurodegenerative disease processes.

## Supporting Information

Figure S1 **SDS-PAGE analysis of purified recombinant TH.** Image of a silver-stained gel in which the left lane was loaded with molecular weight markers and the right lane was overloaded with purified TH.
(TIF)
