# Supplementary figures and images for: BCG Vaccine-Induced Neuroprotection in a Mouse Model of Parkinson's Disease
Source: PLoS One. 2011 Jan 31;6(1):e16610. doi: 10.1371/journal.pone.0016610 (PMC3031604; doi:10.1371/journal.pone.0016610)

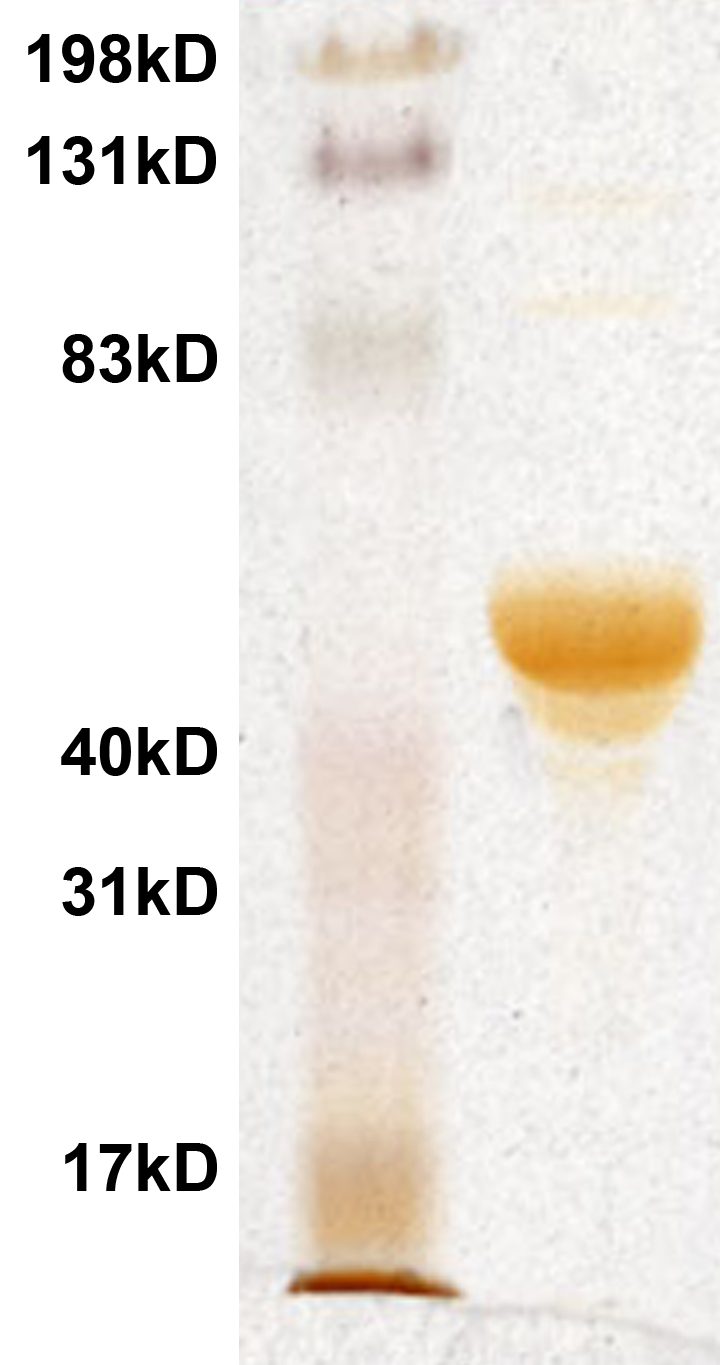

Supplement: Figure S1 — SDS-PAGE analysis of purified recombinant TH. Image of a silver-stained gel in which the left lane was loaded with molecular weight markers and the right lane was overloaded with purified TH. (TIF) [file pone.0016610.s001.tif]
